# Risk of Bacterial Transmission in Bronchiectasis Outpatient Clinics

**DOI:** 10.1007/s13665-018-0203-6

**Published:** 2018-07-02

**Authors:** Philip Mitchelmore, Catherine Wilson, David Hettle

**Affiliations:** 1 Heart and Lung Unit, Torbay Hospital, Torquay, UK; 2 Institute of Biomedical and Clinical Sciences, University of Exeter Medical School, Exeter, UK

**Keywords:** Bronchiectasis, Cross-infection, Infection control, Cystic fibrosis, Bacterial infection, Bacterial transmission

## Abstract

**Purpose of Review:**

The purpose of this review is to discuss the risk of bacterial cross-infection for bronchiectasis patients in the outpatient setting. Cross-infection has primarily been a matter of concern in cystic fibrosis (CF). There is considerable evidence of transmission of pathogens between CF patients, and this has led to guideline recommendations advocating strict segregation policies. Guidelines in bronchiectasis do not specifically address the issue of cross-infection. If cross-infection is prevalent, it may have significant implications for patients and the practical running of specialist care.

**Recent Findings:**

Multiple UK-based studies have now published evidence of cross-infection with *Pseudomonas aeruginosa* within cohorts of bronchiectasis patients; however, the risk does not appear to be high. There is also evidence suggesting cross-infection from CF patients to bronchiectasis patients.

**Summary:**

The current evidence for cross-infection in bronchiectasis is limited, but suggests a small risk with *Pseudomonas aeruginosa*. Longitudinal studies looking at *Pseudomonas aeruginosa* and other pathogens are now required.

## Introduction

The clinical entity of non-cystic fibrosis bronchiectasis, hereafter referred to as simply bronchiectasis, has been recognised since at least the nineteenth century when it was described by Rene Laënnec [1]. Today, a diagnosis of bronchiectasis requires abnormally dilated bronchi on radiological examination, a clinical presentation of a cough with sputum production and recurrent respiratory infections and the exclusion of cystic fibrosis (CF). Bronchiectasis had previously been seen as an orphan disease of decreasing relevance [2]; however, this is no longer the case [3, 4, 5•]. The establishment of registries, such as EMBARC in Europe and the Bronchiectasis and NTM Research Registry in North America, have highlighted the renewed interest in this condition. While global estimates of prevalence are highly variable, it is also likely that current figures underestimate the true disease burden. Despite heightened awareness, a diagnosis can be significantly delayed or wrongly labelled as purely chronic obstructive pulmonary disease [6–8].

**Fig. 1:**
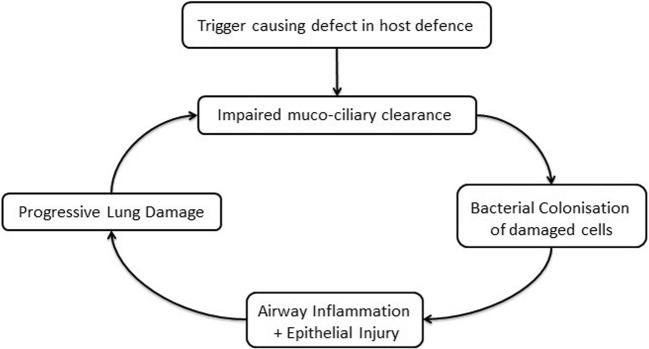
The vicious cycle hypothesis based on Peter Cole’s original description

The principles of managing bronchiectasis continue to be based around the vicious cycle hypothesis set out by Peter Cole (see Fig. 1) [9–11]. A key part of this management strategy is dealing with the bacteria colonising these patients. Certain pathogens are consistently found in microbiological studies of bronchiectasis and include *Pseudomonas aeruginosa*, *Haemophilus influenzae*, *Staphylococcus aureus*, *Streptococcus pneumoniae* and *Moraxella catarrhalis* [12–15]. A further growing concern involves the non-tuberculous mycobacteria (NTM). Colonisation with pathogens is associated with disease severity, with *P. aeruginosa* being particularly implicated [12, 16]. Thus, it would seem logical that avoidance of colonisation by these pathogens would be advantageous. After a number of studies have suggested cross-infection of pathogens between patients in cystic fibrosis cohorts, more recent work has started to explore this issue in bronchiectasis. In this review article, we consider the risk of patients with bronchiectasis acquiring these pathogens in the outpatient setting from other patients.

## Cross-Infection in Cystic Fibrosis—a Reason for Concern in Bronchiectasis

The suspicion of cross-infection in bronchiectasis is unsurprising given the evidence from the CF community concerning patient-to-patient transmission of the *Burkholderia cepacia* complex and *P. aeruginosa*. Evidence that cross-infection was occurring became apparent from studies involving patients: in frequent close contact [17, 18]; in social proximity within holiday camps [17, 19–23]; and within clinical environments [24–28]. Infamous strains associated with increased morbidity and mortality were described, such as the ET12 strain of *Burkholderia cenocepacia* and the Liverpool epidemic strain of *P. aeruginosa* [29–31]. Cross-infection was not a universal finding in epidemiological studies, yet the practice of CF holiday camps was ceased and rigorous infection control policies were introduced into clinical settings [32, 33]. Current examples include the cohorting of clinics by colonising pathogen, preventing direct contact between patients and rotating staff rather than patients through consultation rooms. The demonstrated benefits of these policies have included delayed chronic infection, stopping the spread of a common strain, and reduced prevalence of a transmissible strain [34–37].

A new major concern of cross-infection in the CF community has subsequently arisen. Whole genome sequencing of *Mycobacterium abscessus* isolates has suggested possible cross-infection [38, 39]. This is particularly concerning as the transmissibility of this pathogen would have emerged in the CF community despite segregation and other infection control policies.

## “Proving” Cross-Infection

While the research in the CF community has claimed cross-infection, it is exceedingly difficult to prove. In reality, what is assessed is the likelihood of cross-infection, as opposed to the acquisition of pathogens from the environment. A variety of factors need to be considered for a reliable risk assessment.

An essential starting point for considering cross-infection is examining the evidence of shared strains between patients. However, the identification of a shared strain can be technique dependent [40]. From the early cross-infection studies in the CF community, through to current whole genome sequencing-based work, a wide range of techniques have been used with varying degrees of capability and robustness. These methods can be broadly split into the molecular fingerprinting techniques generally seen in older studies and sequencing-based techniques that are more common in modern studies.

Molecular fingerprinting techniques, such as pulsed-field gel electrophoresis (PFGE) and random amplified polymorphic DNA (RAPD), often involve the amplification of DNA by polymerase chain reaction (PCR) and the subsequent separation of products on a gel. This allows visual examination, either by eye or computer, to identify sufficiently similar patterns as the same strain. However, these techniques are susceptible to interpretive bias and poor inter-laboratory reproducibility.

Sequencing techniques are increasingly common and include whole genome sequencing, but also the simpler multi-locus sequence typing (MLST), a technique which sequences only a few housekeeping genes, with the outputs entered into a global database. This is a highly reproducible scheme with straightforward naming and subsequent identification of shared strains. This scheme also allows easy strain identification from whole genome sequencing data as these housekeeper genes will already have been sequenced. However, MLST is clearly not as detailed as whole genome sequencing as far less genetic material is analysed.

The mere identification of a shared strain by a robust technique is insufficient to prove cross-infection. It is clear from epidemiological studies that some strains of bacteria are common in patients and the general environment, such as the *P. aeruginosa* strain known as Clone C [41, 42]. Consequently, when two patients share a previously described transmissible strain not found in the environment, it is more likely that cross-infection has occurred rather than environmental acquisition, as opposed to patients sharing a strain like Clone C which is known to be common in the environment. Therefore, knowledge of both the shared strain and its environmental prevalence is important.

Further evidence that increases the likelihood of cross-infection is a plausible acquisition route. In general, it is felt that human-to-human transfer of respiratory pathogens takes place by direct and indirect contact transmission, droplet transmission and airborne transmission [32, 43]. Droplet transmission generally refers to particles greater than 5 μm in diameter which do not remain airborne, as opposed to airborne droplet nuclei, which are smaller than 5 μm and can be inhaled [44]. Cross-infection with droplet nuclei is of particular concern. Knibbs et al. demonstrated that cough aerosols containing viable *P. aeruginosa* could travel at least 4 m and be detected in air after 45 min [43]. This time period was consistent with a previous aerobiological model of viable *P. aeruginosa* [45]. An older study found evidence of a transmissible strain via air sampling 1–3 h after patients left their ward room. The same strain of *P. aeruginosa* was also found when sampling ward corridors, spirometry tubing and chairs after use by patients known to be colonised with it [46]. It is possible that survival in aerosols may be enhanced in strains expressing a mucoid phenotype—a common finding in chronic CF strains [45].

Reviewing CF studies, it appeared that by solely implementing measures against contact and droplet transmission, halting the spread of transmissible strains was still not achieved without strict segregation [47, 48]. This adds weight to concerns of airborne transmission via droplet nuclei being a major factor in cross-infection. Consequently, it is conceivable that patients may never physically meet yet may cross-infect. While epidemiological studies cannot identify every occasion where patients have shared an environment, it may be possible to identify some temporal relationship. For example, in previous CF studies, cross-infection may have occurred during holiday camp attendance, while in recent bronchiectasis studies, this could have been during attendance at an outpatient clinic or a pulmonary rehabilitation course.

Finally, understanding the behaviour and potential mutation rates of the bacteria contributes to the assessment of transmission risk. This understanding is potentially critical when using whole genome sequencing data. An issue with whole genome sequencing in cross-infection studies is determining when genetic difference between samples is significant. This is particularly important when assessing whether cross-infection has occurred in respiratory cohorts under long-term follow-up. The longer that patients have been colonised with a particular pathogen, the greater the plausible genetic divergence in potential cross-infection cases. Significant divergence within an individual patient’s lung is also possible [49••], and when transmitted to the lungs of another patient, these pathogens may show even further genetic divergence when subjected to different pressures. Examples of pressures include the use of a long-term inhaled antibiotic or the presence of hypermutator genes. Previous work has illustrated that hypermutators are not uncommon in diseased lungs [50]. Consequently, an understanding of standard mutation rates, the awareness of and testing for hypermutators and an approximation of how long the patient may have had the strain may all influence the likelihood of cross-infection.

It is clear that we need to think in terms of risk and likelihood when considering cross-infection in respiratory cohorts with chronic colonisation. There are many facets to these considerations and with our current knowledge and technologies, these assessments have changed from those of the early CF studies.

## Cross-Infection in Bronchiectasis

As previously mentioned, whilst the investigation of cross-infection is a long-running narrative in CF, it is only starting to be addressed in bronchiectasis. After considering cross-infection in CF, the same issues in bronchiectasis cohorts are clearly plausible. Without guidelines advising segregation, patients will either come into direct contact, or at least share facilities within a short time period. In the outpatient setting, this could include shared waiting areas; the use of rooms for lung function testing and consultations with healthcare professionals; and patients passing through a hospital pharmacy or café. A higher risk environment may be pulmonary rehabilitation courses, where patients may spend many hours together whilst performing exercise. As specialist outpatient attendance and pulmonary rehabilitation is key to good disease management [51, 52], it is important to have an appreciation of the risk of cross-infection. In the last 5 years, there have been three particularly relevant pieces of research which begin to quantify that risk, though all three solely address *P. aeruginosa* (see Table 1) [49••, 53••, 54••].

**Table 1:** Summary of studies suggesting evidence of cross-infection with *P. aeruginosa* in bronchiectasis

Authors	Sample sizes	Outpatient setting	Genotyping techniques	Likelihood of cross-infection
De Soyza A et al. *Eur Respir J. 2014* [53••]	40 patients 56 isolates	Single-centre CF managed on different site	- ArrayTube genotyping - Variable number tandem repeat (VNTR) analysis - Pulsed-field gel electrophoresis	“Only one probable case of cross-infection”
Hilliam Y et al. *Eur Respir J. 2017* [49••]	91 patients 189 isolates	Multi-centre (16 “non-CF bronchiectasis” centres)	- Whole genome sequencing	Closely related isolates found between patients “implying the possible occurrence of cross-infection”
Mitchelmore PJ et al. *Thorax. 2017* [54••]	46 patients 459 isolates	Single-centre CF managed on same site	- Random amplification of polymorphic DNA - Multi-locus sequence typing - Whole genome sequencing	A shared strain identified between three patients had little genetic difference. Believed to be “indicative of cross-infection”

The first of these, published by De Soyza et al., was an epidemiological review of *P. aeruginosa* in an outpatient setting in the North-East of England [53••]. Of the 40 patients studied, 36 were seen within a specialist bronchiectasis outpatient service. The authors reported that cross-infection almost certainly occurred between two patients. This study was pivotal as it was the first to report evidence that cross-infection in the outpatient setting may be occurring between bronchiectasis patients. Crucially, if it had occurred, it did not appear to be a common event. Two separate genotyping techniques were used for robustness on all samples, with additional analysis with a further technique (PFGE) in the case of possible cross-infection. It is worth pointing out that the bronchiectasis service was run on a different site to the local CF services, and as the authors comment, this may have influenced the findings. In other settings, CF and bronchiectasis patients may use the same clinic rooms and see the same healthcare providers. Also of note in this study, there is no reporting of multiple isolates being assessed from each sputum sample. It is clearly not feasible to assess all the colonies seen on a bacterial culture plate; however, this may result in an underestimation of the presence of shared strains.

A contrasting study was published by Hilliam et al. in which whole genome sequencing data was presented from 91 patients attending 16 different bronchiectasis centres [49••]. This provided a useful overview of the strains of *P. aeruginosa* common in the UK and highlighted that ubiquitous environmental strains were found in patients, suggesting common environmental acquisition. This multi-centre study was less able to comment on local cross-infection; however, there were cases of closely related bacterial isolates from different patients within the same centre. It is of some re-assurance that there was no suggestion of a widespread, nationally transmissible strain, though the lack of genetic diversity between some isolates highlights the possibility that cross-infection could be occurring. The study also demonstrated that patients can be infected with multiple bacterial lineages. This re-enforces how analysing single isolates from culture samples may risk missing additional strains.

The final study of note examined a single-centre cohort of 46 bronchiectasis patients in the South-West of England [54••]. The study included three genotyping techniques (RAPD, MLST and whole genome sequencing) assessing 10 isolates per sample, and investigated historical microbiological data and hospital attendance. Attempts were made to further interpret genetic differences in shared strains via an *in silico* prediction of hypermutator status and by incorporating publicly available genomes of the shared strains into the analysis. Multiple shared strains were found and the majority were felt to be due to environmental acquisition. However, one shared strain was identified (ST564), which has rarely been described in the environment, and was highly genetically similar between three patients. An episode when two of the three patients shared facilities could be identified, and it was felt that cross-infection was highly likely. It should be noted that when considering previous microbiological data, this event may have been a case of super-infection. To highlight the influence of higher resolution genotyping in risk assessment, it is worth noting this cohort had previously been presented in abstract form after RAPD and MLST had been performed [55], and the perceived likelihood of cross-infection having occurred increased after the addition of whole genome sequencing analysis. This study also assessed the local CF population as well as non-respiratory isolates and ST564 was not found elsewhere. There was also no evidence of cross-infection between the bronchiectasis and CF cohorts.

This publication, as well as the other two main studies, lacks longitudinal data which would help give more context to the importance of this potentially transmissible strain. The studies also lack epidemiological data for pathogens in the outpatient environment, such as from the water supplies. Consequently, we do not have evidence, either for or against, of acquisition from the outpatient facilities.

The authors of the final study suggest that a change in segregation policy is not recommended. This is following consideration of both the likely rare occurrence of cross-infection, and the potential disadvantages of this strict approach. However, it may be the case that the risk is greater in situations where bronchiectasis patients share facilities with CF patients. A case report describes highly likely transmission of *P. aeruginosa* to a bronchiectasis patient who had shared accommodation and physiotherapy with CF patients [56], and another series suggests that patients with bronchiectasis had been potentially infected with transmissible strains of *P. aeruginosa* from CF patients during inpatient admissions [57•]. Despite the absence of reported transmission of other bacteria between CF and NCFB patients (notably, NTM, *S. aureus* or *H. influenzae*), it would seem sensible that bronchiectasis patients who are managed within a CF service should be subject to the same infection control measures in order to reduce the risk of transmission. This is consistent with a recent working group consensus statement involving prominent European networks and a patient group [58••].

## Future Research Needs

The possibility of cross-infection between bronchiectasis patients in the outpatient setting is clearly plausible and is a research concern for the bronchiectasis community [59]. At present, there is insufficient evidence of a significant risk to patients, which may be due to the limited quantity of relevant work. Currently, only cross-infection with *P. aeruginosa* has been investigated but this has been in small numbers and compounded by a lack of longitudinal data. The rates of *P. aeruginosa* colonisation are lower in bronchiectasis patients than in CF [53••], and hospital attendances are typically shorter. Therefore, if there was a transmissible strain within a cohort, it would likely take longer to reveal itself, and follow up data would require an adequate interval. Longitudinal studies would also help to clarify whether or not putative transmissible strains persist. Taking things forward, large longitudinal studies with high-resolution genotyping and detailed epidemiological data collection are required for *P. aeruginosa*, as well as other significant pathogens. In light of the increasing incidence and prevalence of non-tuberculous mycobacterial-pulmonary disease and concerns in the CF community of NTM cross-infection [38, 39, 60], in certain circumstances, these studies should include *M. abscessus*.

If future work reveals evidence of transmissible pathogens, further research should look into strain pathogenicity, examine infection control policies such as segregation or face-mask wearing and ultimately investigate the clinical impact of infection control policies.

Although performing these studies is clearly important, gaining a better understanding of molecular techniques and data interpretation is also essential. This may focus around whole genome sequencing and the interpretation of genetic difference between samples. We are not yet able to clearly define the significance of genetic difference; therefore, an understanding of divergence is crucial.

## Conclusion

At present, the evidence for the risk of cross-infection in the outpatient setting is very limited. There is evidence of likely cross-infection with *P. aeruginosa*, although these episodes seem to be rare. With potentially growing cohorts, and the promotion of bronchiectasis-specific clinics and pulmonary rehabilitation programmes, further high-quality research is required to investigate cross-infection risk by *P. aeruginosa* and other pathogens. With our current knowledge base, adherence to sensible basic infection control measures should be standard practice, without the imposition of stricter segregation policies [58••].
